# The Role of Parental and Adolescent Psychosocial Factors in Different Aggression Profiles: A Comparative Approach

**DOI:** 10.3390/jcm14061924

**Published:** 2025-03-12

**Authors:** Mimma Tafà, Luca Cerniglia, Silvia Cimino

**Affiliations:** 1 Department of Clinical, Dynamic and Health Psychology, Sapienza, University of Rome, 00185 Rome, Italy; mimma.tafa@uniroma1.it (M.T.); silvia.cimino@uniroma1.it (S.C.); 2 Faculty of Psychology, International Telematic University Uninettuno, 00186 Rome, Italy

**Keywords:** parental dysregulation, adolescents’ fights, psychopathology, family dynamics, couple conflict

## Abstract

**Background:** This study explores parental dysregulation when associated with adolescents’ involvement in street fights with peers. Parental dysregulation, characterized by emotional volatility, impulsivity, and inconsistent discipline, significantly affects adolescent development, influencing their social, emotional, and behavioral functioning. Street fights, which involve physical violence among adolescents in public settings, pose risks to both individuals and the community. This research aims to identify risk factors and underlying mechanisms associated with adolescent street fights, providing insights for targeted interventions and prevention strategies. **Aim:** The study employs social learning theory to explain how adolescents may model aggressive behaviors observed in dysregulated parents and family systems theory to highlight the role of dysfunctional family dynamics in being associated with aggression. A sample of 292 male adolescents and their parents was assessed using self-report measures. **Results:** Statistical analyses revealed higher levels of emotional dysregulation, depression, and hostility among parents of adolescents frequently involved in street fights. **Conclusions:** These findings underscore the need for interventions focusing on improving parental emotional regulation, reducing hostile behaviors, and enhancing family communication to mitigate adolescent aggression. Further research should explore diverse populations and longitudinal data to strengthen these conclusions.

## 1. Introduction

Parental dysregulation refers to the difficulties parents experience in regulating their emotions and behaviors [1,2]. It encompasses a range of dysregulated responses, including emotional volatility, impulsivity, and inconsistent discipline [3]. Parental dysregulation has been found to have significant implications for various aspects of adolescent development, including social, emotional, and behavioral functioning [4,5]. One important area of adolescent development that is influenced by parental dysregulation is their engagement in street fights with peers. In social learning theory [6], observational learning suggests that adolescents exposed to physical conflict at home are more likely to resort to physical aggression in peer interactions, rather than verbal or covert aggression [7]. Moreover, street fights often occur in low-supervision public settings, where adolescents who experience dysfunctional family dynamics may seek peer validation through dominance and retaliation [8]. Street fights are aggressive encounters that take place in public settings, involving physical violence between adolescents [9]. These fights can have detrimental consequences for the individuals involved, as well as the broader community [10]. Studying the relationship between parental dysregulation and adolescents’ engagement in street fights is significant for several reasons. First, it helps to identify risk factors associated with aggressive behavior among adolescents, providing insights into the underlying mechanisms that contribute to street fights. Second, understanding this relationship can inform the development of targeted interventions and prevention strategies to reduce aggression and promote peaceful peer interactions [11]. By addressing parental dysregulation, it could be possible to mitigate the occurrence of street fights and promote healthier forms of conflict resolution among adolescents.

Social learning theory posits that individuals learn behaviors, including aggression, through observation and imitation of others [12]. In the context of parental dysregulation and adolescent engagement in street fights, this theory suggests that adolescents may acquire and reinforce aggressive behaviors if they observe and model their parents’ dysregulated responses. Parental dysregulation may serve as a powerful socializing factor, shaping the beliefs, attitudes, and behavioral tendencies of adolescents [13,14,15]. Research has demonstrated the influence of parental dysregulation on the acquisition and reinforcement of aggressive behaviors among adolescents. For example, a study by Han et al. [3] found that parental negative emotionality and emotional dysregulation were associated with increased aggression in children.

Furthermore, family systems theory emphasizes the interdependence and interconnectedness of family members within a larger system [16]. In the context of adolescent engagement in street fights, this theory highlights the role of family dynamics, conflict, and parental modeling. Dysfunctional family dynamics characterized by high levels of conflict, poor communication, and ineffective problem-solving strategies can contribute to the development of aggressive behaviors in adolescents [17]. In fact, one of the major advances in the literature on family relationships from the past decade has been the emphasis on family strengths in being associated with offspring well-being. In particular, family conflict and parental depression can significantly influence depression in adolescents and, in particular, the former can contribute to its transmission between generations [18].

This study, in a scientific panorama lacking in longitudinal research, examined the associations between family conflict and depressive symptoms of adolescents and parents, measured at three points in the relationship. 

In particular, it emerges that family conflict plays a critical role in adolescents’ depressive symptoms and in their intergenerational transmission; however, it is particularly maternal depressive symptoms that influence those of adolescent children.

More generally, the findings highlight the fundamental role of family dynamics in mental health, especially in the development of adolescent depressive symptoms. Interventions aimed at reducing family conflict can help attenuate depressive symptoms across generations.

Parental modeling of aggression, particularly in the form of physical discipline or violent conflict resolution strategies, can also shape adolescents’ engagement in street fights [19]. Research has shown that family factors, such as parental conflict and violence, are associated with increased aggression and involvement in street fights among adolescents [20]. Moreover, parental psychopathology has been posited to be associated with maladaptive behavioral and emotional functioning in offspring and specifically with mothers’ and fathers’ dysregulation and aggressive conduct in youths [21,22,23]. Depression and hostility in adults, even at subclinical levels, have been associated with youths’ difficulty emotionally self-soothing and modulating their emotions (e.g., [24,25]).

Other experts underlined the importance of peer socialization, which refers to the influence of peers on individual behavior, attitudes, and values [26]. In the context of parental dysregulation and engagement in street fights, peer socialization plays an important role. Peers can reinforce or discourage aggressive behaviors, depending on the norms and values within their social group [27]. The interaction between parental dysregulation and peer processes is complex and bidirectional. For example, adolescents with dysregulated parents may be more likely to seek out peers who engage in aggressive behaviors, leading to a reinforcement of their own aggressive tendencies [28].

After reviewing the existing research and theoretical frameworks, this study will evaluate possible differences in the scores of dysregulation, and depressive and hostility symptoms in parents of adolescents who are frequently involved in fights with peers (according to the definition of [29]). The study will also assess possible differences in the scores of perceived filial self-efficacy and the quality of their relationship with peers in their adolescent offspring.

## 2. Materials and Methods

### 2.1. Sample and Procedure

A consecutive snowball sampling approach was adopted in collaboration with public and private schools to form a convenience sample from the general population. Our selection criteria for the enrollment of adolescents and their parents consisted of the following: (1) absence of diagnosed psychiatric conditions among the participants and (2) no ongoing medical or psychological treatments. In total, we enrolled 329 male adolescents aged between 14 and 17 years and their parents. Given that the study sample consists of male adolescents, it is important to note that boys are more likely to engage in overt physical aggression compared to girls, who tend to exhibit relational aggression [30,31]). We excluded individuals who declined to participate (*n* = 18), those whose parents refused consent (N = 15), and those with a psychiatric diagnosis (*n* = 4). Consequently, the final sample size for this study comprised 292 male adolescents and their parents (N_tot_ = 876). The majority of the participants were Caucasian (97.3%), and 84% of their families had an annual household income ranging from EUR 28,000 to 55,000. Among the adolescents, 88.6% came from intact family structures. The authors affirm that all procedures adhered to the ethical standards outlined by relevant national and institutional committees on human experimentation and were in compliance with the 2008 revision of the Helsinki Declaration of 1975. The research plan for this study was approved by the Ethical Committee of La Sapienza, University of Rome (Approval No. 0023/22), prior to its commencement. Written informed consent was obtained from all participants’ parents or guardians, ensuring the privacy and anonymity of personal information. During school hours, under the supervision of a member of the research team and a teacher, youths completed a series of self-report measures. Parents filled out the measures at home. Following a previous study (see De Looze et al., 2012 [32] for details), adolescents were asked to fill out an ad hoc designed questionnaire to distinguish individuals who engage in habitual violence from those who display occasional or no violent behavior. Based on their responses, the adolescents were divided into two groups: Group 1 consisted of individuals reporting less than four fights in the previous year, while Group 2 included those reporting four or more fights. Demographic characteristics of these groups are presented in Table 1.

### 2.2. Measures

#### 2.2.1. Emotion Regulation

To measure emotion regulation, the Difficulties in Emotion Regulation Scale ([32]; DERS) was used. The DERS is a 36-item self-report questionnaire that assesses the six dimensions of emotion regulation: (a) lack of emotional awareness; (b) lack of emotional clarity; (c) impulse control difficulties; (d) difficulties in engaging in goal-directed behaviors); (e) non-acceptance of emotional responses; and (f) limited access to emotion regulation strategies. The items are rated in terms of how frequently each statement applies to them on a five-point Likert scale ranging from 1 (“almost never”) to 5 (“almost always”). The total score for the DERS is calculated by adding all answers and the subscale scores are also added to a total number. The DERS has demonstrated high internal consistency, with a Cronbach’s α of 0.93. In the current study, Cronbach’s alpha was 0.92 for the total scale for adolescents, 0.75 for awareness, 0.82 for clarity, 0.86 for impulsivity, 0.85 for goals, 0.86 for non-acceptance, and 0.82 for strategies.

#### 2.2.2. Parental Psychopathological Symptoms

Psychopathological symptoms in mothers and fathers were assessed by the SCL-90/R [33], a 90-item self-report symptom inventory measuring psychopathological symptoms and psychological distress rated on a Likert scale of 0 (not at all) to 4 (extremely). It asks subjects to report if they have suffered in the past week from symptoms of somatization (e.g., headaches), obsessive-compulsivity (e.g., having to check and double-check what you do), interpersonal sensitivity (e.g., feeling that people are unfriendly or dislike you), depression (e.g., feeling blue), anxiety scale (e.g., feeling fearful), hostility (e.g., having urges to beat, injure, or harm someone), phobic anxiety (e.g., feeling afraid to go out of your house alone), paranoid ideation (e.g., persecutory beliefs concerning a perceived threat toward oneself), and psychoticism (e.g., having thoughts that are not your own). Aside from these nine primary scales, the questionnaire provides a global severity index (GSI). In this study, only the subscales of Depression and Hostility have been considered, and they both have shown a high internal consistency (Cronbach’s alpha = 0.87).

#### 2.2.3. The Quality of Their Relationships with Peers

Adolescents completed the Inventory of Peer Attachment [34]. The scale measures how much adolescents feel they can trust, communicate with, and are supported by their peers. Using a five-point scale (1 = almost never to 5 = almost always), adolescents answered nine questions about their friends in the past month (e.g., ‘I trusted my friends’, ‘I could count on my friends when I needed to talk’, ‘My friends showed that they understand me’). For each wave, the nine items were averaged. A measure of peer support was created by taking the sum of the two waves (range = 1–10), such that higher scores indicated greater peer support. The scale had good internal consistency (α = 0.84).

#### 2.2.4. Perceived Filial Self-Efficacy

The questionnaire assesses filial perception of their parents’ accessibility, sensitivity and support in day-to-day situations and in hypothetical critical moments in adolescents’ lives. Higher scores in this questionnaire indicate higher perceived support from parents [35]. Adolescents’ perceived filial self-efficacy (PFSE) was measured by 16 items rated on a 7-point scale from ‘‘strongly disagree” (scored as 1) to ‘‘strongly agree” (scored as 7) assessing belief in their capabilities to discuss with their parents personal problems even under difficult circumstances; to cultivate positive affective ties and manage negative emotional reactions toward them; to get parents to see their side on contentious issues; manage stress arising from parents’ marital conflicts; and to influence constructively parental attitudes and social practices. ‘‘I can get my parents to understand my point of view when it differs from theirs” is an item measuring a sense of efficacy in managing potentially contentious issues. The construction of the scale was guided by knowledge concerning prototypic situations adolescents have to manage with their parents (Italian version, [36]). It had a reliability coefficient of 0.87 [6].

#### 2.2.5. Analysis Plan

All analyses were performed using SPSS software, Version 26 (IBM Corp. Released 2019. IBM SPSS Statistics for Windows, Version 26.0. Armonk, NY: IBM Corp). Before conducting ANOVA, key assumptions were tested. The Shapiro–Wilk test indicated that the data were approximately normally distributed (*p* = 0.05). Levene’s test for equality of variances was non-significant (*p* = 0.05), confirming the homogeneity of variances across groups. Boxplots did not reveal extreme outliers that could bias the results. These findings confirm that the assumptions for ANOVA were met. ANOVAs for repeated assessments were used to compare the outcomes on all variables for Group 1 and Group 2. *p* values less than <0.001 were considered significant. The mean values include the SDs. Cohen’s (1988) criteria were followed, and a power analysis of 0.86. A post hoc power analysis was conducted using GPower [37] to determine whether the sample size was sufficient to detect significant differences in parental dysfunction and psychosocial variables across aggression levels. Given a total sample size, an alpha level of 0.05, and a medium effect size (Cohen’s d = 0.50 or η² = 0.06), the achieved statistical power was 0.86, meeting the conventional standard for adequate power [38]. This suggests that the study had sufficient sensitivity to detect moderate effects of parental dysfunction on adolescent aggression. As the study used different sources for data collection (e.g., parental vs. adolescent reports), the likelihood of Common Method Bias was reduced.

## 3. Results

An ANOVA between Group 1 (families with youths reporting less than four fights in the previous year) and Group 2 (families with youths reporting four or more fights in the previous year) was performed regarding maternal and paternal subscales of dysregulation, depression, and hostility, and adolescents’ dysregulation, perceived filial self-efficacy, and the quality of their relationships with peers. Notably, the analyses showed significant differences in all of the subscales evaluated in this study. Specifically, mothers and fathers in Group 2 showed significantly higher (i.e., more maladaptive) scores on the dysregulation, depression, and hostility scales when compared with parents belonging to Group 1. Moreover, adolescents of Group 2 shower higher (i.e., more maladaptive) scores in dysregulation, perceived filial self-efficacy, and the quality of their relationships with peers. The largest differences between the two groups were on the depression and hostility scales. In particular, significant and marked differences were found on the hostility scale in fathers (higher in fathers belonging to Group 2), and on the depression scale in mothers (again, higher in fathers belonging to Group 2) (Table 2).

## 4. Discussion

The study found that parents of adolescents who engaged in frequent street fights (Group 2) exhibited significantly higher levels of emotional dysregulation, depression, and hostility compared to parents of adolescents who engaged in fewer fights (Group 1). These findings align with the social learning theory, which posits that children learn behaviors through observation and imitation of their parents. Adolescents may model the dysregulated emotional responses and aggressive behaviors observed in their parents, leading to an increased propensity for violent encounters with peers [39]. This underscores the importance of addressing parental dysregulation as a potential mechanism for reducing aggressive behaviors in adolescents [15]. While the study found that parents of adolescents who engaged in frequent street fights exhibited significantly higher levels of emotional dysregulation, depression, and hostility, it is important to consider previous contrasting evidence that suggests the relationship between parental dysregulation and adolescent aggression might be moderated by other factors. Margolin and Baucom [7], for example, found that although parental physical aggression was indeed associated with adolescent aggression, the direct effect of emotional dysregulation was less clear. Their study highlighted that peer influence and the adolescent’s own emotional regulation strategies significantly impacted whether parental aggression translated to aggressive behavior in adolescents. This indicates that external factors beyond parental dysregulation need to be considered when addressing adolescent aggression. Family systems theory also supports these findings, suggesting that dysfunctional family dynamics, characterized by high conflict and poor communication, contribute to the development of aggressive behaviors in adolescents [16,40]. The higher scores on the depression and hostility scales among parents in Group 2 reflect a more maladaptive family environment, which likely exacerbates adolescents’ aggressive tendencies. Parental depression and hostility have been shown to have significant adverse effects on adolescent behavior. For instance, Franck and Buehler [41] demonstrated that marital hostility and parental depressive affect are conjoint familial stressors that contribute to adolescent problem behaviors. Their study found that marital hostility was directly associated with externalizing problems in adolescents, while parental depressive affect was linked to both internalizing and externalizing problems.

Further supporting this, a study by Knox et al. [42] highlighted that parental hostility is a more likely associated factor with children’s current and future aggression and conduct problems than parental depression. This finding suggests that the emotional environment created by hostile parental behaviors significantly shapes adolescent aggression. The link between parental mental health and adolescent aggression is further reinforced by Knox et al. [42], who found that maternal aggression was associated with changes in amygdala resting-state functional connectivity, which mediated the onset of major depressive disorder in adolescents. This neurobiological pathway highlights how parental aggression can influence the emotional and behavioral regulation of adolescents, leading to increased aggression.

## 5. Conclusions

The findings suggest that interventions aimed at reducing adolescent aggression should consider the family context, particularly parental emotional regulation and behavior. Programs that focus on improving parents’ emotional regulation skills, reducing depressive and hostile behaviors, and fostering better communication and problem-solving strategies within the family could be effective in mitigating adolescents’ aggressive behaviors. Additionally, enhancing adolescents’ perceived filial self-efficacy and improving the quality of their peer relationships could further support these efforts, promoting healthier conflict resolution strategies and reducing the likelihood of engagement in street fights.

While the study provides valuable insights, it is important to acknowledge its limitations. The sample was predominantly Caucasian and from intact families, which may limit the generalizability of the findings to more diverse populations. Future research should aim to include more diverse samples to examine whether these findings hold across different cultural and family contexts. Additionally, only self-report measures were used to assess the study variables. Furthermore, longitudinal studies would help to better understand the causal relationships between parental dysregulation, family dynamics, and adolescent aggression.

## Figures and Tables

**Table 1:** Demographic characteristics of subjects in Group 1 and Group 2.

	Group 1	Group 2	Significance
Participants	192	100	-
Age (mean [SD])	16.5	16.9	NS
Household income (mean)	31,000 euros	32,000 euros	NS
Intact families	88.8%	87.7%	NS

NS = not significant (*p* > 0.05).

**Table 2:** Mean scores and standard deviation of all study variables.

	Group 1	Group 2	η^2^
Fathers	M (SD)	M (SD)	
Dysregulation	62.33 (10.24)	102.12 (18.45)	0.71 **
Depression	0.62 (0.71)	0.95 (0.63)	0.79 **
Hostility	0.56 (0.46)	0.85 (0.71)	0.81 **
Mothers			
Dysregulation	65.13 (12.52)	104.27 (19.72)	0.72 **
Depression	0.62 (0.71)	0.92 (0.44)	0.88 **
Hostility	0.64 (0.62)	0.95 (0.73)	0.69 **
Adolescents			
Dysregulation	62.36 (13.75)	103.63 (18.31)	0.68 **
Perceived Filial Self-efficacy	4.34 (1.22)	2.64 (1.44)	0.71 **
Quality of Their Relationship with Peers	3.44 (1.03)	2.11 (1.32)	0.69 **

Note. η^2^: eta-squared. ** *p* < 0.001.

## Data Availability

Data will be available through reasonable requests to the authors.
